# Plant-based meat and dairy substitutes on the Norwegian market: comparing macronutrient content in substitutes with equivalent meat and dairy products

**DOI:** 10.1017/jns.2022.6

**Published:** 2022-02-10

**Authors:** Live Edvardsen Tonheim, Elisabeth Austad, Liv Elin Torheim, Sigrun Henjum

**Affiliations:** Department of Nursing and Health Promotion, Faculty of Health Science, Oslo Metropolitan University, 0130 Oslo, Norway

**Keywords:** Dairy substitutes, Macronutrients, Meat substitutes, Milk substitutes, Online stores, Salt content

## Abstract

*Objective:* To assess and compare the macronutrient and salt content in meat and dairy substitutes available on the Norwegian market. *Design*: Comparison between substitute products and two groups of meat and dairy products where one group represented the healthiest option (Keyhole) and one the most used option (Regular). Kruskal–Wallis test with pairwise comparison was conducted on categories with more than two groups, and Mann–Whitney *U* test was conducted on categories with two groups. *Setting*: Online stores in Norway. Hundred and two meat substitute products and 173 milk and dairy substitute products on sale spring and autumn 2020 were assessed; additionally, ninety-eight equivalent meat products and 105 milk and dairy products. *Results*: While Keyhole and Regular meat did not contain fibre, meat substitutes contained 3⋅5–5⋅0 g fibre per 100 g. The saturated fat content in meat substitutes was on average 1⋅9 times lower than in Keyhole products and 5⋅8 times lower than in Regular products. Milk and dairy substitutes contained 3⋅2 and 3⋅4 times less protein than Keyhole and Regular products, respectively. *Conclusions*: The study results indicate that meat and dairy substitutes on the Norwegian market vary in nutritional composition. Compared to Keyhole and Regular, substitutes contained lower levels of saturated fat, meat substitutes contained higher levels of fibre and milk and dairy substitutes less protein. Future studies should include content of micronutrients for a more comprehensive assessment.

## Introduction

A shift towards more plant-based diets is named as one of the measures needed to combat both climate change and non-communicable diseases (NCDs)^(1,2)^. Livestock production alone is responsible for about 14 % of global anthropogenic greenhouse gas emissions^(3)^. In addition, animal-source foods require more environmental resources in terms of fresh water and land use compared with plant-based foods^(1,4)^. Given the predicted population growth and increased affluence of low- and middle-income countries, the global demand for animal-source foods is expected to surpass the planet's capacity in 2050^(5–7)^.

Unhealthy or inadequate diets are the leading causes of death and loss of healthy life years worldwide^(8)^. According to the Global Burden of Disease study, 11 million deaths by NCDs are diet-related^(8)^. In Norway, an unhealthy diet is one of the three main risk factors for premature mortality^(9)^. Although healthy diets can be composed in many different ways, there is general agreement that the consumption of processed and red meats should be limited^(10)^, and the Norwegian dietary guidelines recommend a maximum of 500 g per week^(11)^. There is equally strong evidence of the health benefits of wholegrain, fruits, non-starchy vegetables and pulses/legumes. However, the place of dairy products in a healthy diet is debated^(12)^. In Norway, a daily intake of low-fat dairy is recommended, while a reduction in whole-fat dairy products is advised^(11)^. Further recommendations include choosing wholegrain, increasing the amounts of fruits, berries and vegetables, and limiting the intake of sugar, salt and saturated fat. To help Scandinavian consumers make healthy food choices, the Keyhole front-of-pack label is used to identify options with a more beneficial nutrient profile (less saturated fat, salt and sugar, and more dietary fibre) in a given category^(13)^. The Keyhole is a voluntary endorsement scheme, similar to the ‘Choices logo’ used in Poland and the Czech Republic, and the ‘Heart Symbol’ used in Finland^(13,14)^. As a simple labelling scheme, it provides information about the total product, not nutrient content, as the more complex ‘UK Multiple Traffic Lights’ or the Australasian ‘Health Star Rating’^(14)^.

As in most Western countries, Norwegian meat consumption has steadily increased since the 1970s^(15)^. Consumption of cheese and yoghurt also continues to rise, whereas demand for milk is decreasing. Nevertheless, in recent years, flexitarian eating (cutting down on meat and other animal-source foods on a regular basis) has become more common^(16)^. Simultaneously, consumer demand for plant-based products substituting meat and dairy, such as plant-based ‘milk’, burgers and sausages, has increased^(17,18)^. Consumer concerns about health and sustainability are suggested as one of the drivers for this increasing demand^(19)^. In a Norwegian study on meat-free habits, a clear majority (71 %) named health as the main reason for wanting to reduce meat consumption^(16)^. Another motivation was concern for the environment. Plant-based eating is associated with being ‘healthy’ and ‘sustainable’^(20)^. This might contribute to plant-based substitutes being uncritically, and possibly wrongly, perceived as healthy and sustainable.

Plant-based meat substitutes have been available for many years, but mainly marketed to vegetarians and vegans^(21)^. The early meat substitutes were mostly made from soy protein and subjected to a low grade of processing, i.e. tofu and tempeh^(22)^. Gradually, however, technological developments have resulted in products intended to mimic meat in taste and texture^(23)^. This new generation of plant-based meat analogues (PBMA) is based not only on soy, but also on protein isolates from peas, wheat, chickpeas, beans and fungi^(17,24)^. The increasing selection of non-dairy milk substitute products seems to appeal to cow's milk consumers as well as to vegetarians and vegans or to people with intolerances or allergies^(25)^. In addition to plant-based milk, a wide range of other non-dairy products is available, including plant-based alternatives for cheese, yoghurts, ice cream and cream^(12)^.

Although plant-based substitutes may provide a higher intake of recommended food groups compared with their animal counterparts, the nutrient composition of these products can vary widely^(24,26,27)^. Meat substitutes are suggested to contain more fibre and less total and saturated fat compared with their meat counterparts^(26)^. Furthermore, these products have been found to contain comparable amounts of protein and iron^(22)^, but higher amounts of salt^(26)^. The nutritional properties of plant-based milk alternatives have also been found to vary, depending on plant source and fortification with micronutrients such as calcium, B12 and iodine in particular^(27–29)^. Mäkinen *et al.,* therefore, highlight the importance for manufacturers to consider nutrient content and quality if these products are to be marketed as substitutes for cow's milk^(27)^.

As the appeal of plant-based meat and dairy substitutes spreads to a wider population, the potential consequences for public health need to be addressed^(17)^. Little is known about the nutritional composition of meat and dairy substitute products on sale in Norway. Moreover, no specific regulations governing the nutrient content of these products are currently in place. The main object of this explorative study is, therefore, to evaluate the macronutrient composition of meat and dairy substitutes available on the Norwegian market, and compare them with their animal-based analogues.

## Materials and methods

### Store selection

Mapping was conducted of plant-based meat and dairy substitute products on sale in three Norwegian online grocery stores between March and April 2020. The three stores (kolonial.no, meny.no and matlevering.coop.no) were selected because they represent retailers holding the majority of the total grocery market share in Norway, NorgesGruppen (44 %), Coop (29 %) and Rema 1000 (23 %)^(30)^. Special health food or vegan online stores were excluded because these stores are not commonly used by the general public. In addition, two grocery stores were physically visited. Due to COVID-19 control measures, the stores had to be situated nearby the researcher collecting the data, and the two largest grocery stores in close proximity to the researcher were thus chosen. Tightened restrictions also prevented further stores to be visited. Therefore, the remainder of the data collection, including the supplementary search carried out between 1 October and 16 October, was conducted exclusively on online stores. The two searches were carried out in connection with the spring and autumn 2020 release of new products.

### Product selection

All available plant-based substitute products in the visited online and physical stores, including those temporarily sold out, were registered in a database.

Animal-based samples were selected based on the same usability as their plant-based counterparts. Best comparable usability was defined by product type, product name and description, flavour and texture. Furthermore, two sets of criteria for the inclusion of counterparts were used to form two different groups for comparison purposes. The first group, representing the healthiest animal-based options, only included products carrying the Keyhole label^(13)^. The other group, representing commonly used products, included well-known brands in addition to the three retailer's own brands. ‘Most common’ was defined as most popular according to ‘sort by popularity’ in two of three online websites and/or available in all three online stores. In this group, the exclusion criteria were the Keyhole label and additional flavouring that did not match the plant-based comparable option. In categories where the number of suitable counterparts was high, the most common products with the best comparable usability were selected. In cases where the number of products was high, a decision on which products to include was made by the project group. If available, an animal-based equivalent from the same producer as the plant-based product was chosen. In some cases, nutritional composition data were unavailable. These products were included in the database but excluded from the statistical analysis.

### Data entry

The final database contained 102 meat substitute products and 173 milk and dairy substitute products (Table 1). A total of ninety-eight meat products, twenty-eight of which carried the Keyhole label and seventy of which were selected as commonly used products, were registered as meat counterparts. Similarly, 105 milk and dairy products, including 15 carrying the Keyhole label and 90 representing commonly used products, were registered. Plant-based meat substitutes and meat counterparts were categorised as burgers, sausages, mince, meatballs, nuggets and schnitzels and cold cuts. Each category comprised a minimum of five products. The remaining products constituted a residual category. Due to heterogeneity in product type and usability, this category was excluded from statistical analysis. Milk and dairy substitutes and counterparts were categorised as creams and crème fraiche, cheese, flavoured drinks and iced coffee, milk, ice creams and yoghurts. In the cheese category, where identical products were available in both blocks and slices, ‘slices’ were excluded from the statistical analysis. Other products excluded from the dairy categories were yoghurts with granola or muesli. Nutrition composition data were obtained from the producer's website if available. Otherwise, information on nutrient content was retrieved from one of the online stores or the website of a distributor at www.askoservering.no/. The nutrition declaration provided by the food industry was considered reliable^(31)^. Nutrient content of total fat, saturated fat, carbohydrates, sugars, proteins, fibre and salt for all products was reported in grams, milligrams or micrograms per 100 grams. Information collected on energy was reported in kilojoules and kilocalories. For products identified in both the main and supplementary searches, nutritional information from the main search was used. All records of nutritional information were checked against the information on the websites by two researchers in the team. In cases where discrepancies between the recorded values and the information on the websites were detected, values likely due to typing errors were changed, and values likely due to changes in recipes were retained.

**Table 1.** Number of registered substitute products and meat and dairy products according to product categories

Category	Substitute products		Keyhole products	Regular products
	Database	Statistical analysis		
	(*n* 102)	(*n* 82)	(*n* 28)	(*n* 70)
Burgers	21	21	2	18
Sausages	14	14	7	11
Mince	13	13	13	13
Meatballs	10	10	1	10
Nuggets/schnitzel	12	12	2	10
Cold cuts	12	12	3	8
Others	20	0	0	0
	(*n* 173)	(*n* 162)	(*n* 15)	(*n* 90)
Cheese	39	36	5	25
Creams/ crème fraîche	9	9	0	4
Flavoured drinks/ iced coffees	10	10	0	10
Milk (unflavoured drinks)	44	41	10	9
Ice creams	24	24	0	23
Yoghurts	47	42	0	19

### Statistics

Plant-based substitute products for meat (M-Substitutes) were compared with Keyhole-labelled meat (M-Keyhole) and ‘Regular’ meat (M-Regular). Similarly, plant-based products for dairy (D-Substitutes) were compared with Keyhole-labelled dairy products (D-Keyhole) and ‘Regular’ dairy products (D-Regular). Data were reported as the median nutrient content of macronutrients and salt measured in grams per 100 grams of product and energy percentage (E%) in the three groups. The normality of data distribution was tested through the Shapiro–Wilk test and visual examination of QQ-plots, and rejected. Sub-categories with three groups (Substitutes, Keyhole and Regular) were compared using the Kruskal–Wallis test with pairwise comparisons. In the sub-categories that did not include Keyhole-labelled products (creams and crème fraiche, flavoured drinks and iced coffees, ice creams and yoghurts), comparisons were made between D-Substitutes and D-Regular using the Mann–Whitney *U* test. In all tests, a *P*-value below 0⋅05 was regarded significant. All analyses were conducted in IBM SPSS statistics 28 (IBM Corp., Armonk, NY, USA).

## Results

The total sample of products included six sub-categories: eighty-two M-Substitutes, twenty-eight M-Keyhole and M-Regular, all from six sub-categories (Table 1). Likewise, from six sub-categories: 162 D-Substitutes, 15 D-Keyhole and 90 D-Regular.

### Meat substitute products and meat products

Overall, M-Substitute products contained more fibre than both M-Keyhole and M-Regular products (both *P* < 0⋅001) (Table 2) (Table 5 and 6 in supplements). In all but one category (cold cuts), median fibre content per 100 g ranged from 3⋅5 to 5⋅0 g in M-Substitute products, whereas the median fibre content in both meat groups was 0⋅0 g (Table 3). When comparing all products, median saturated fat content in M-Substitutes was 2 times lower than in M-Keyhole (*P* < 0⋅001) and 5⋅8 times lower than in M-Regular (*P* < 0⋅001) (Table 2). Similar differences in saturated fat content were observed in all sub-categories between M-Substitutes and M-Regular (Table 3), but not between M-Substitutes and M-Keyhole. The only difference in salt content was found in mince, where M-Substitutes contained ten times more salt than M-Keyhole (*P* = 0⋅001), and five times more than M-Regular (*P* = 0⋅010) (Table 3). Overall, protein content was lower in M-Substitutes than in both M-Keyhole (*P* < 0⋅001) and M-Regular (*P* = 0⋅007) (Table 2). However, only the sub-categories burgers and mince had significantly different protein content (Table 3). In burgers, M-Substitutes contained less protein than both M-Keyhole (*P* = 0⋅007) and M-Regular (*P* = 0⋅002). In mince, M-Substitutes contained less protein than M-Keyhole (*P* = 0⋅010). As expected, the overall carbohydrate content was higher in M-Substitutes than in both meat groups (both *P* < 0⋅001), but differed only in the sub-categories, burgers and mince (Table 3). M-Substitutes contained more carbohydrates than both M-Keyhole (burgers: *P* = 0⋅005, mince: *P* < 0⋅001) and M-Regular (burgers: *P* < 0⋅001, mince: *P* < 0⋅001).

**Table 2.** Differences in nutrient content between all plant-based substitute products for meat and dairy, meat and dairy products with keyhole and meat dairy products without keyhole per 100 grams of product

All meat substitute products and meat, median (IQR)							
Nutrients per 100 g	Substitutes (*n* 82)		Keyhole (*n* 28)		Regular (*n* 70)		*P*-value*
Energy (kcal)	201⋅5	(169⋅5–220⋅5)^a^	145⋅0	(125⋅0–155⋅8)^b^	224⋅5	(204⋅8–244⋅3)^c^	<0⋅001
Fat (g)	10⋅2	(8⋅1–15⋅0)^a^	7⋅2	(5⋅0–9⋅0)^b^	16⋅5	(14⋅0–18⋅1)^c^	<0⋅001
Saturated fat (g)	1⋅1	(0⋅8–1⋅7)^b^	2⋅3	(2⋅0–3⋅1)^b^	6⋅4	(5⋅6–7⋅7)^a^	<0⋅001
Carbohydrates (g)	8⋅4	(4⋅9–12⋅6)^a^	0⋅1	(0⋅0–5⋅5)^b^	4⋅0	(0⋅0–7⋅1)^b^	<0⋅001
Sugars (g)	1⋅0	(0⋅6–1⋅7)^a^	0⋅0	(0⋅0–0⋅4)^b^	0⋅2	(0⋅0–1⋅0)^c^	<0⋅001
Fibre (g)	3⋅6	(0⋅0–5⋅1)^a^	0⋅0	(0⋅0–0⋅0)^b^	0⋅0	(0⋅0–0⋅0)^b^	<0⋅001
Protein (g)	13⋅0	(8⋅3–16⋅3)^a^	18⋅0	(11⋅5–19⋅8)^b^	15⋅0	(11⋅3–17⋅7)^b^	<0⋅001
Salt (g)	1⋅5	(1⋅1–1⋅8)	1⋅0	(0⋅1–1⋅7)	1⋅5	(1⋅0–1⋅7)	0⋅081
All dairy substitute products and dairy with three groups of comparison, median (IQR)							
Nutrients per 100 g	Substitutes (*n* 77)		Keyhole (*n* 15)		Regular (*n* 34)		*P*-value*
Energy (kcal)	62⋅0	(40⋅0–270⋅0)^b^	38⋅0	(33⋅0–260⋅0)^b^	277⋅0	(65⋅8–339⋅8)^a^	<0⋅001
Fat (g)	3⋅0	(1⋅3–21⋅0)^a^	0⋅5	(0⋅1–16⋅0)^b^	22⋅5	(4⋅0–27⋅0)^c^	<0⋅001
Saturated fat (g)	0⋅6	(0⋅2–18⋅2)^b^	0⋅3	(0⋅1–10⋅0)^b^	15⋅0	(2⋅6–17⋅0)^a^	0⋅001
Carbohydrates (g)	9⋅4	(4⋅3–20⋅0)^a^	4⋅5	(0⋅0–4⋅6)^b^	2⋅7	(0⋅3–4⋅5)^b^	<0⋅001
Sugars (g)	1⋅8	(0⋅0–3⋅9)	4⋅5	(0⋅0–4⋅6)	2⋅0	(0⋅1–4⋅5)	0⋅480
Fibre (g)	0⋅0	(0⋅0–0⋅6)^a^	0⋅0	(0⋅0–0⋅0)^b^	0⋅0	(0⋅0–0⋅0)^b^	<0⋅001
Protein (g)	0⋅5	(0⋅1–1⋅3)^a^	3⋅5	(3⋅5–31⋅0)^b^	11⋅5	(3⋅5–26⋅0)^b^	<0⋅001
Salt (g)	0⋅1	(0⋅1–1⋅9)	0⋅1	(0⋅1–1⋅1)	1⋅1	(0⋅1–1⋅3)	0⋅136
All dairy substitutes products and dairy with two groups of comparison, median (IQR)							
Nutrients per 100 g	Substitutes (*n* 85)				Regular (*n* 56)		*P*-value*
Energy (kcal)	103⋅0	(68⋅0–184⋅0)			124⋅0	(73⋅3–252⋅0)	0⋅117
Fat (g)	7⋅0	(2⋅0–10⋅2)			8⋅3	(2⋅9–13⋅3)	0⋅187
Saturated fat (g)	1⋅7	(0⋅3–7⋅0)			5⋅4	(1⋅8–7⋅8)	0⋅005
Carbohydrates (g)	10⋅0	(6⋅8–23⋅5)			12⋅0	(5⋅7–24⋅0)	0⋅535
Sugars (g)	7⋅5	(3⋅9–18⋅5)			11⋅0	(5⋅7–22⋅4)	0⋅001
Fibre (g)	0⋅8	(0⋅0–1⋅0)			0⋅0	(0⋅0–0⋅0)	<0⋅001
Protein (g)	1⋅4	(1⋅0–3⋅3)			3⋅7	(3⋅5–4⋅4)	<0⋅001
Salt (g)	0⋅1	(0⋅1–0⋅2)			0⋅1	(0⋅1–0⋅2)	0⋅107

IQR, Inter Quartile Range.

^a,b,c^Groups with different superscript differ in Kruskal–Wallis pairwise comparison (*P* <0⋅05).

* Kruskal–Wallis test for differences between three groups of comparison (categories: cheese and milks (unflavoured drinks)); Mann–Whitney *U* test for differences between two groups of comparison (categories: creams/crème fraiche, flavoured drinks/iced coffees, ice creams, yoghurts).

**Table 3.** Differences in nutrient content between plant-based meat substitutes, meat with keyhole and meat without keyhole per 100 g of product, according to burgers, sausages, mince and meatballs sub-categorisation

Burgers, median (IQR)							
Nutrients per 100 g	Substitutes (*n* 21)		Keyhole (*n* 2)^†^		Regular (*n* 18)		*P*-value*
Energy (kcal)	183⋅0	(161⋅5–206⋅0)^b^	127⋅5^b^		225⋅0	(210⋅0–231⋅0)^a^	0⋅002
Fat (g)	9⋅0	(5⋅8–13⋅5)^b^	5⋅8^b^		17⋅3	(15⋅0–18⋅0)^a^	<0⋅001
Saturated fat (g)	0⋅8	(0⋅6–4⋅2)^a^	2⋅1		7⋅8	(6⋅3–8⋅2)^b^	<0⋅001
Carbohydrates (g)	9⋅8	(5⋅9–12⋅5)^a^	0⋅1^b^		1⋅3	(0⋅0–3⋅0)^b^	<0⋅001
Sugars (g)	1⋅3	(0⋅9–2⋅1)^a^	0⋅0^b^		0⋅0	(0⋅0–0⋅1)^b^	<0⋅001
Fibre (g)^‡^	3⋅5	(0⋅0–5⋅3)^a^	0⋅0		0⋅0	(0⋅0–0⋅0)^b^	<0⋅001
Protein (g)	12⋅0	(6⋅9–15⋅5)^a^	19⋅0^b^		15⋅7	(15⋅0–17⋅7)^b^	0⋅001
Salt (g)	1⋅2	(1⋅0–1⋅5)	0⋅8		1⋅0	(1⋅0–1⋅2)	0⋅114
Sausages, median (IQR)							
Nutrients per 100 g	Substitutes (*n* 14)		Keyhole (*n* 7)		Regular (*n* 11)		*P*-value*
Energy (kcal)	198⋅5	(173⋅8–223⋅3)^a^	148⋅0	(146⋅0–156⋅0)^b^	241⋅0	(222⋅0–250⋅0)^c^	<0⋅001
Fat (g)	11⋅5	(10⋅0–16⋅3)^a^	9⋅0	(9⋅0–10⋅0)^b^	18⋅5	(18⋅0–19⋅9)^c^	<0⋅001
Saturated fat (g)	1⋅3	(0⋅9–4⋅7)^b^	3⋅2	(2⋅9–3⋅2)^b^	7⋅1	(6⋅9–7⋅2)^a^	<0⋅001
Carbohydrates (g)	6⋅3	(4⋅4–10⋅3)	5⋅5	(5⋅3–6⋅2)	5⋅6	(5⋅0–6⋅3)	0⋅781
Sugars (g)	1⋅1	(0⋅7–1⋅7)^a^	0⋅5	(0⋅4–0⋅7)^b^	0⋅7	(0⋅4–1⋅3)	0⋅044
Fibre (g)	3⋅5	(0⋅0–4⋅6)^a^	0⋅0	(0⋅0–0⋅0)^b^	0⋅0	(0⋅0–0⋅0)^b^	<0⋅001
Protein (g)	13⋅0	(8⋅3–15⋅4)	11⋅0	(10⋅0–11⋅0)	10⋅0	(9⋅9–14⋅5)	0⋅860
Salt (g)	1⋅8	(1⋅3–1⋅9)	1⋅7	(1⋅7–1⋅8)	1⋅7	(1⋅6–1⋅9)	0⋅954
Mince, median (IQR)							
Nutrients per 100 g	Substitutes (*n* 13)		Keyhole (*n* 13)		Regular (*n* 13)		*P*-value*
Energy (kcal)	195⋅0	(157⋅5–220⋅0)^b^	129⋅0	(120⋅5–146⋅5)^a^	202⋅0	(200⋅0–205⋅0)^b^	<0⋅001
Fat (g)	9⋅6	(4⋅3–13⋅7)^b^	5⋅0	(4⋅9–8⋅3)^b^	14⋅0	(14⋅0–14⋅0)^a^	<0⋅001
Saturated fat (g)	1⋅3	(0⋅5–5⋅5)^b^	2⋅3	(2⋅1–2⋅6)^b^	6⋅3	(6⋅2–7⋅2)^a^	0⋅001
Carbohydrates (g)	5⋅3	(3⋅3–9⋅5)^a^	0⋅0	(0⋅0–0⋅0)^b^	0⋅0	(0⋅0–0⋅0)^b^	<0⋅001
Sugars (g)	0⋅8	(0⋅5–1⋅7)^a^	0⋅0	(0⋅0–0⋅0)^b^	0⋅0	(0⋅0–0⋅0)^b^	<0⋅001
Fibre (g)	4⋅9	(0⋅0–5⋅9)^a^	0⋅0	(0⋅0–0⋅0)^b^	0⋅0	(0⋅0–0⋅0)^b^	<0⋅001
Protein (g)	17⋅3	(14⋅3–19⋅8)^a^	19⋅0	(19⋅0–21⋅5)^b^	19⋅0	(17⋅5–19⋅4)	0⋅031
Salt (g)	1⋅0	(0⋅6–1⋅7)^a^	0⋅1	(0⋅1–1⋅0)^b^	0⋅2	(0⋅1–1⋅0)^b^	0⋅002
Meatballs, median (IQR)							
Nutrients per 100g	Substitutes (*n* 10)		Keyhole (*n* 1)		Regular (*n* 10)		*P*-value*
Energy (kcal)	184⋅5	(167⋅5–214⋅0)	156⋅0	(156⋅0–156⋅0)	205⋅0	(181⋅8–220⋅5)	0⋅191
Fat (g)	9⋅4	(5⋅8–12⋅5)^a^	8⋅7	(8⋅7–8⋅7)	14⋅2	(12⋅8–16⋅0)^b^	0⋅008
Saturated fat (g)	1⋅0	(0⋅7–1⋅2)^a^	2⋅0	(2⋅0–2⋅0)	5⋅6	(3⋅7–6⋅2)^b^	<0⋅001
Carbohydrates (g)	10⋅0	(6⋅2–14⋅4)	4⋅9	(4⋅9–4⋅9)	4⋅9	(4⋅2–6⋅8)	0⋅067
Sugars (g)	1⋅1	(0⋅9–2⋅0)	0⋅4	(0⋅4–0⋅4)	1⋅0	(0⋅2–1⋅4)	0⋅322
Fibre (g)	4⋅5	(0⋅0–5⋅3)	0⋅0	(0⋅0–0⋅0)	0⋅0	(0⋅0–0⋅4)	0⋅063
Protein (g)	13⋅7	(8⋅1–18⋅1)	14⋅0	(14⋅0–14⋅0)	13⋅5	(10⋅8–14⋅9)	0⋅955
Salt (g)	1⋅5	(1⋅1–1⋅6)	1⋅7	(1⋅7–1⋅7)	1⋅8	(1⋅6–1⋅9)	0⋅111

IQR, Inter Quartile Range.

^a,b,c^Groups with different superscript differ in Kruskal–Wallis pairwise comparison (*P* < 0⋅05).

* Kruskal–Wallis test for differences between groups.

† IQR missing due to *n* = 2.

‡ Differences between Keyhole and Regular in percentiles outside IQR.

### Milk and dairy substitute products and milk and dairy

Combined D-Substitutes for milk and cheese had seven times lower protein content than D-Keyhole (*P* < 0⋅001) and twenty-three times lower than D-Regular (*P* < 0⋅001) (Table 2). Overall, lower protein content in D-substitutes than in D-Regular was also found in creams/crème fraiche, favoured drinks/iced coffees, ice creams and yoghurts (*P* < 0⋅001). Lower protein content in D-Substitutes than D-Keyhole and D-Regular was also found across all sub-categories (Table 4 and Table 7 in supplements). D-Substitutes for milk and cheese had an overall higher carbohydrate content than D-Keyhole and D-Regular (both *P* < 0⋅001) (Table 2). However, the overall carbohydrate content in the remaining categories did not differ between D-Substitutes and D-Regular. The saturated fat content in both D-Substitutes and D-Keyhole in combined analysis for milk and cheese was substantially lower than in D-Regular (*P* = 0⋅003, *P* < 0⋅001, respectively) (Table 2). However, no significant difference was found in the combined analysis of creams/crème fraiche, favoured drinks/iced coffees, ice creams and yoghurts, and there were wide variations between sub-categories (Tables 2 and 4). In cheese, the median saturated fat content in D-Substitutes was 1⋅9 times higher than in D-Keyhole (*P* = 0⋅003), but no difference was found in comparison with D-Regular. The opposite applied to milk, where both D-Substitutes and D-Keyhole had four times lower saturated fat content than D-Regular (both *P* < 0⋅001). No difference in saturated fat content was observed in yoghurts and ice creams. However, D-Substitutes in flavoured drinks/iced coffees contained less saturated fat than D-Regular (*P* < 0⋅001). Salt content differed only in cheese, where D-Substitutes contained 1⋅8 times more salt than D-Keyhole (*P* = 0⋅012) and 1⋅7 times more salt than D-Regular (*P* < 0⋅001).

**Table 4.** Differences in nutrient content between plant-based substitute products, dairy with keyhole and dairy without keyhole per 100 grams of product, according to sub-categories cheese, milk (unflavoured drinks) and yoghurts

Cheese, median (IQR)							
Nutrients per 100 g	Substitutes (*n* 36)		Keyhole (*n* 5)		Regular (*n* 25)		*P*-value*
Energy (kcal)	270⋅0	(227⋅3–285⋅0)^b^	268⋅0	(237⋅0–270⋅0)^b^	300⋅0	(257⋅0–351⋅0)^a^	0⋅006
Fat (g)	21⋅0	(20⋅0–23⋅0)^a^	16⋅0	(13⋅0–16⋅0)^b^	26⋅0	(21⋅0–27⋅0)^c^	<0⋅001
Saturated fat (g)	18⋅7	(10⋅3–21⋅0)^b^	10⋅0	(8⋅0–10⋅5)^a^	17⋅0	(14⋅0–17⋅0)^b^	0⋅010
Carbohydrates (g)	20⋅0	(9⋅9–21⋅8)^a^	0⋅0	(0⋅0–0⋅0)^b^	1⋅0	(0⋅1–2⋅7)^b^	<0⋅001
Sugars (g)	0⋅0	(0⋅0–0⋅5)	0⋅0	(0⋅0–0⋅0)^a^	0⋅3	(0⋅0–2⋅7)^b^	0⋅015
Fibre (g)	0⋅0	(0⋅0–0⋅0)	0⋅0	(0⋅0–0⋅0)	0⋅0	(0⋅0–0⋅0)	0⋅392
Protein (g)	0⋅2	(0⋅0–0⋅7)^a^	31⋅0	(30⋅0–31⋅5)^b^	24⋅0	(6⋅3–27⋅0)^b^	<0⋅001
Salt (g)	2⋅0	(1⋅5–2⋅3)^a^	1⋅1	(1⋅1–1⋅3)^b^	1⋅2	(1⋅0–1⋅4)^b^	<0⋅001
Milks (unflavoured drinks), median (IQR)							
Nutrients per 100 g	Substitutes (*n* 41)		Keyhole (*n* 10)		Regular (*n* 9)		*P*-value*
Energy (kcal)	42⋅0	(30⋅0–54⋅0)^b^	36⋅5	(33⋅0–38⋅0)^b^	43⋅0	(41⋅0–65⋅0)^a^	0⋅012
Fat (g)	1⋅3	(1⋅1–1⋅9)^b^	0⋅3	(0⋅1–0⋅6)^a^	1⋅2	(1⋅0–4⋅0)^b^	<0⋅001
Saturated fat (g)	0⋅2	(0⋅1–0⋅3)^b^	0⋅2	(0⋅1–0⋅3)^b^	0⋅8	(0⋅7–2⋅6)^a^	<0⋅001
Carbohydrates (g)	5⋅6	(2⋅4–7⋅9)	4⋅5	(4⋅5–4⋅6)	4⋅5	(4⋅5–4⋅8)	0⋅898
Sugars (g)	2⋅9	(2⋅0–4⋅8)^a^	4⋅5	(4⋅5–4⋅6)^b^	4⋅5	(4⋅5–4⋅8)	0⋅027
Fibre (g)	0⋅5	(0⋅0–0⋅7)^a^	0⋅0	(0⋅0–0⋅0)^b^	0⋅0	(0⋅0–0⋅0)^b^	<0⋅001
Protein (g)	0⋅7	(0⋅4–1⋅7)^a^	3⋅5	(3⋅4–3⋅5)^b^	3⋅4	(3⋅3–3⋅5)^b^	<0⋅001
Salt (g)	0⋅1	(0⋅1–0⋅1)	0⋅1	(0⋅1–0⋅1)	0⋅1	(0⋅1–0⋅1)	0⋅911
Yoghurts, median (IQR)							
Nutrients per 100 g	Substitutes (*n* 42)				Regular (*n* 19)		*P*-value*
Energy (kcal)	80⋅0	(67⋅5–107⋅3)			86⋅0	(71⋅0–90⋅0)	0⋅379
Fat (g)	2⋅5	(2⋅0–7⋅7)			3⋅0	(3⋅0–3⋅4)	0⋅345
Saturated fat (g)	0⋅5	(0⋅3–5⋅7)			2⋅0	(1⋅9–2⋅3)	0⋅070
Carbohydrates (g)	8⋅9	(6⋅5–12⋅0)			11⋅0	(5⋅6–12⋅0)	0⋅731
Sugars (g)	6⋅8	(2⋅6–8⋅1)			10⋅0	(5⋅6–11⋅0)	0⋅001
Fibre (g)	1⋅0	(0⋅6–1⋅0)			0⋅0	(0⋅0–0⋅0)	<0⋅001
Protein (g)	1⋅7	(1⋅1–3⋅7)			4⋅1	(3⋅7–5)	<0⋅001
Salt (g)	0⋅1	(0⋅0–0⋅2)			0⋅1	(0⋅1–0⋅1)	0⋅826

IQR, Inter Quartile Range.

^a,b,c^Groups with different superscript differ in Kruskal–Wallis pairwise comparison (*P* < 0⋅05).

* Kruskal–Wallis test for differences between three groups of comparison (categories: cheese and milks (unflavoured drinks)); Mann–Whitney *U* test for differences between two groups of comparison (categories: creams/crème fraiche, flavoured drinks/iced coffees, ice creams, yoghurts).

## Discussion

To the best of our knowledge, the present study is the first in Norway to compare the available selection of meat and dairy substitute products with animal-based products. Overall results indicated that both meat substitutes and Keyhole-labelled meat have a lower content of saturated fat and higher content of fibre than Regular meat. However, the protein content in the dairy substitutes was considerably lower than in the Keyhole-labelled and Regular dairy products.

### Meat substitutes

In agreement with previous studies, meat substitute products had a higher fibre content than Regular meat products^(22,26)^. As meat do not contain fibre, and many substitute products are based on plant foods that are rich in fibre^(26)^, these results were as expected. There is a general consensus that increased intake of dietary fibre can have health benefits such as lower risk of hypertension, stroke, obesity and certain gastrointestinal conditions and diseases^(32)^. Since an increased intake of fibre may reduce cholesterol and high blood pressure^(32)^, it is advised to increase the fibre intake in the Norwegian population. The intake of dietary fibre in the Norwegian population is currently lower than the recommended 3 g per megajoule^(15)^. Although, it is the whole composition of the diet that ultimately determines the total intake of fibre, replacing meat for meat substitutes may contribute to increase overall fibre in the diet. However, there is insufficient knowledge about whether nutrients in processed products offer the same health benefits as the whole foods on which they are based^(17)^.

Although debated, reduction of saturated fat in the diet has been found to reduce risk of cardiometabolic diseases^(33,34)^. The World Health Organization recommends limiting intake of saturated fat for a healthy diet^(35)^, and in Norway, the population goal is to keep saturated fat below 10 % of total energy intake^(36,37)^. As meat is one of the main sources of saturated fat, reducing intake of red and processed meat products is recommended^(11)^. The present study found the overall content of saturated fat in plant-based meat substitutes to be almost six times lower than the overall content in Regular meat, and the findings were consistent across all categories. This may suggest that replacing Regular meat products for plant-based products could contribute to reduction of saturated fat intake in the diet. Furthermore, the requirements for producers to apply the Keyhole label include limitations in saturated fat content. Thus, similar or lower content of saturated fat found in substitutes compared to Keyhole-labelled meat, further indicate that meat substitutes may be a healthier choice in terms of saturated fat. These results were consistent with a similar Australian study^(26)^ and may suggest a potential health benefit from choosing meat substitutes over meat. However, another study comparing nutrient content in meat substitutes with meat products observed equal levels of saturated fat in burgers^(22)^.

### Milk and dairy substitutes

Milk and dairy substitutes in the present study contained considerably lower levels of protein compared with products based on cow's milk and were observed in all sub-categories. Although, similar trends have been found in previous studies assessing both milk^(25,28)^ and cheese substitutes^(38)^, protein content may vary both between- and within-product categories^(38–40)^. Craig and Fresán analysed the nutrient content of plant-based beverages according to the type of beverage, and found high median protein content (8–9 g/100 g) in beverages based on soy, peas or combinations of legumes and nuts/grains, and low median protein content in (0⋅1–1⋅3 g/100 g) beverages based on nuts, almonds or rice^(40)^.

As expected, milk and dairy substitutes seemed overall to have a more beneficial saturated fat content compared with regular milk and dairy products, though there were wide variations between the different sub-categories. Previous studies have found fatty acid profiles to vary according to product's plant source, and in dairy substitutes, milks and cheese based on coconut have been found to have the highest content of saturated fats^(12,22,25,28,38)^. It was beyond the scope of this study to investigate nutrient composition according to natural ingredients. Nevertheless, the content of coconut fat may be a possible explanation for the median saturated fat content in cheese substitutes, which was close to twice the amount of Keyhole-labelled cheese, and slightly higher than in Regular cheese. Considering the soaring demand and availability of coconut-based substitute products, especially in the dairy categories, the potential impact on nutrient intake should be addressed.

Although the overall salt content did not differ between plant-based milk and dairy substitute products, cheese substitutes contained almost twice as much salt as Keyhole-labelled cheese. These findings may indicate that consumer should be aware of salt levels in some product categories of plant-based substitute.

### Strengths and limitations

The strength of the present study was that the substitute products were compared with both Regular and Keyhole-labelled meat and dairy options. This allowed for a comparison of plant-based substitutes with both the commonly consumed and the healthiest available products. Using Keyhole-labelled products gave the further advantage of including counterparts already complying standardised and known criteria for healthier products. As health is one of the main reasons for choosing a more plant-based diet, this was a valuable comparison. However, a major limitation of the present study was that only macronutrient content was investigated. Animal-based foods are an important source of a range of essential micronutrients, such as iron and zinc, and the only source of B12^(41,42)^. An Australian study found meat substitute products to contain less B12, iron and zinc compared with equivalent meat varieties^(26)^. Cow's milk and dairy products are important sources of iodine and calcium^(43,44)^. Unless fortified, plant-based alternatives either do not contain these nutrients or contain insignificant amounts^(12,28)^. This is, of concern, partly because iodine deficiency has re-emerged as a public health problem in Norway, especially among women of reproductive age^(45)^. Young women are also found to be most likely to shift their diet towards more plant-based eating, and might therefore be more susceptible to plant-based milk and dairy alternatives^(16,46)^. Substitute products may provide a culturally and socially convenient path towards a more plant-based diet^(47)^. However, Salome *et al.* found that the nutritional impact of substitute products in the diet vary, and depend on both the products that are replaced and the substitutes used as replacement^(48)^.

Another limitation in the present study was that the range of regular meat and dairy products was not included. Furthermore, inclusion was not based on real sales numbers, and the criteria aiming to include the most used products resulted in smaller sample of regular meat and dairy products than plant-based substitutes. Furthermore, in some categories, there were only one or two Keyhole-labelled products, and in others, Keyhole-labelled products were not in sale. In the former case, results from the comparisons between substitutes and Keyhole-labelled products could be questioned due to lack of counterparts. Notably, the criteria for the Keyhole label heavily limit the content of nutrients such as sugar, salt and saturated fat. Thus, some product categories may fail to meet the criteria, due to being unhealthy (e.g. ice cream). Other possible explanation for the lack of available Keyhole-labelled products in some sub-categories could be that producers may not wish to make the necessary changes in their products’ recipes to meet the criteria, or they have not applied for approval under the Keyhole labelling scheme even though their products meet the criteria.

## Conclusion

In summary, the study results indicate that meat and dairy substitutes on the Norwegian market vary in nutritional composition. Compared to Keyhole and Regular, substitutes contained lower levels of saturated fat, meat substitutes contained higher levels fibre and milk- and dairy substitutes less protein. However, as the market for plant-based substitutes continues to grow, a comprehensive evaluation of the nutritional composition, including micronutrients and possible health benefits and risks is needed.

## Supplementary material

For supplementary material accompanying this paper visit https://doi.org/10.1017/jns.2022.6. click here to view supplementary material
