# Supplementary material for: Plant-based meat and dairy substitutes on the Norwegian market: comparing macronutrient content in substitutes with equivalent meat and dairy products
Source: J Nutr Sci. 2022 Feb 10;11:e9. doi: 10.1017/jns.2022.6 (PMC8889083; doi:10.1017/jns.2022.6)
Supplement: Supplementary file 1 [file S2048679022000064sup001.docx]

Table 5 Differences in nutrient content between all plant-based substitute products for meat and dairy, meat and dairy products with
keyhole and meat dairy products without keyhole per 100 g of product

| **All meat substitute products and meat, median (IQR)** | | | | | | | | | | |
| --- | --- | --- | --- | --- | --- | --- | --- | --- | --- | --- |
| **Nutrients per 100 g** | **Substitutes (n=82)** | | | **Keyhole (n=28)** | | | **Regular (n=70)** | | | **P-value*** |
| Energy (kcal) | 201.5 | (169.5-220.5)^a^ | | 145.0 | (125.0-155.8)^b^ | | 224.5 | (204.8-244.3)^c^ | | <0.001 |
| Fat (E%) | 49.5 | (40.0-62.4)^a^ | | 40.8 | (35.1-54.6)^b^ | | 66.9 | (62.4-70.1)^c^ | | <0.001 |
| Saturated fat (E%) | 4.8 | (3.9-7.0)^a^ | | 16.6 | (12.2-19.0)^b^ | | 26.3 | (23.6-30.6)^c^ | | <0.001 |
| Carbohydrates (E%) | 17.3 | (10.1-28.1)^a^ | | 0.2 | (0.0-14.9)^b^ | | 6.9 | (0.0-12.6)^b^ | | <0.001 |
| Sugars (E%) | 2.2 | (1.2-3.4)^a^ | | 0.0 | (0.0-1.1)^b^ | | 0.4 | (0.0-1.8)^b^ | | <0.001 |
| Fibre (E%) | 3.7 | (0.0-5.5)^a^ | | 0.0 | (0.0-0.0)^b^ | | 0.0 | (0.0-0.0)^b^ | | <0.001 |
| Protein (E%) | 25.8 | (16.3-33.7)^b^ | | 48.4 | (31.3-64.3)^a^ | | 25.0 | (19.7-31.5)^b^ | | <0.001 |
| Salt (g) | 1.5 | (1.1-1.8) | | 1.0 | (0.1-1.7) | | 1.5 | (1.0-1.7) | | 0.081 |
|  |  |  | |  |  | |  |  | |  |
| **All dairy substitute products and dairy with three groups of comparison, median (IQR)** | | | | | | | | | | |
| **Nutrients per 100 g** | **Substitutes (n=77)** | | | **Keyhole (n=15)** | | | **Regular (n=34)** | | | **P-value*** |
| Energy (kcal) | 62.0 | (40.0-270.0)^b^ | | 38.0 | (33.0-260.0)^b^ | | 277.0 | (65.8-339.8)^a^ | | <0.001 |
| Fat (E%) | 53.2 | (36.6-72.6)^b^ | | 12.5 | (2.7-52.9)^a^ | | 67.7 | (55.4-70.2)^b^ | | <0.001 |
| Saturated fat (E%) | 14.4 | (4.5-63.3)^b^ | | 7.5 | (2.7-33.1)^b^ | | 43.6 | (36.0-50.8)^a^ | | 0.002 |
| Carbohydrates (E%) | 31.1 | (20.8-55.0)^b^ | | 48.6 | (0.0-54.5^b^ | | 3.8 | (0.3-27.1)^a^ | | <0.001 |
| Sugars (E%) | 6.6 | (0.0-31.6) | | 48.6 | (0.0-54.5) | | 3.2 | (0.1-27.1) | | 0.054 |
| Fibre (E%) | 0.0 | (0.0-2.6)^a^ | | 0.0 | (0.0-0.0)^b^ | | 0.0 | (0.0-0.0)^b^ | | <0.001 |
| Protein (E%) | 3.3 | (0.3-9.0)^a^ | | 42.4 | (37.8-46.3)^b^ | | 29.9 | (19.9-31.7)^c^ | | <0.001 |
| Salt (g) | 0.1 | (0.1-1.9) | | 0.1 | (0.1-1.1) | | 1.1 | (0.1-1.3) | | 0.136 |
|  |  |  |  |  |  |  |  |  |  |  |
| **All dairy substitutes products and dairy with two groups of comparison, median (IQR)** | | | | | | | | | | |
| **Nutrients per 100g** | **Substitutes (n=85)** | | |  |  |  | **Regular (n=56)** | | | **P-value*** |
| Energy (kcal) | 103.0 | (68.0-184.0) | |  |  |  | 124.0 | (73.3-252.0) | | 0.117 |
| Fat (E%) | 42.3 | (27.2-58.6) | |  |  |  | 41.7 | (30.0-52.9) | | 0.752 |
| Saturated fat (E%) | 14.9 | (4.4-32.9) | |  |  |  | 27.2 | (19.5-33.7) | | 0.006 |
| Carbohydrates (E%) | 45.5 | (30.7-58.4) | |  |  |  | 46.7 | (33.7-51.8) | | 0.619 |
| Sugars (E%) | 35.3 | (15.0-46.2) | |  |  |  | 43.3 | (32.3-47.6) | | 0.012 |
| Fibre (E%) | 1.4 | (0.0-2.8) | |  |  |  | 0.0 | (0.0-0.0) | | <0.001 |
| Protein (E%) | 5.2 | (2.8-11.7) | |  |  |  | 11.8 | (6.9-20.9) | | <0.001 |
| Salt (g) | 0.1 | (0.1-0.2) | |  |  |  | 0.1 | (0.1-0.2) | | 0.107 |

IQR, Inter Quartile Range
^a,b,c^ Groups with different superscript differ in Kruskal-Wallis pairwise comparison (p <0.05)
* Kruskal-Wallis Test for differences between three groups of comparison (categories: cheese and milks (unflavoured drinks));
Mann-Whitney U Test for differences between two groups of comparison (categories: creams/crème fraiche, flavoured drinks/iced coffees,
ice creams, yoghurts)

Table 6 Differences in nutrient content between plant-based meat substitutes, meat with keyhole and meat without keyhole per 100 g
of product, according to burgers, sausages, mince and meatballs sub-categorization.

| **Burgers, median (IQR)** | | | | | | | | | | | |
| --- | --- | --- | --- | --- | --- | --- | --- | --- | --- | --- | --- |
| **Nutrients per 100 g** | **Substitutes (n=21)** | | **Keyhole (n=2)** ^†^ | | | | **Regular (n=18)** | | | | **P-value*** |
| Energy (kcal) | 183.0 | (161.5-206.0)^b^ | 127.5^b^ |  |  | | 225.0 | | (210.0-231.0)^a^ | | 0.002 |
| Fat (E%) | 42.6 | (34.9-55.2)^b^ | 40.5^b^ |  |  | | 69.2 | | (64.9-70.4)^a^ | | <0.001 |
| Saturated fat (E%) | 4.4 | (3.2-17.3)^a^ | 14.9 |  |  | | 31.1 | | (28.1-32.0)^b^ | | <0.001 |
| Carbohydrates (E%) | 24.4 | (12.1-36.2)^a^ | 0.2^b^ |  |  | | 2.1 | | (0.0-5.4)^b^ | | <0.001 |
| Sugars (E%) | 2.5 | (1.7-5.7)^a^ | 0.0^b^ |  |  | | 0.0 | | (0.0-0.2)^b^ | | <0.001 |
| Fibre (E%) ^‡^ | 4.1 | (0.0-6.4)^a^ | 0.0 |  |  | | 0.0 | | (0.0-0.0)^b^ | | <0.001 |
| Protein (E%) | 26.9 | (20.7-33.3)^a^ | 59.7^b^ |  |  | | 28.9 | | (26.0-32.2) | | 0.030 |
| Salt (g) | 1.2 | (1.0-1.5) | 0.8 |  |  | | 1.0 | | (1.0-1.2) | | 0.114 |
|  |  |  |  |  |  | |  | |  | |  |
| **Sausages, median (IQR)** | | | | | | | | | | | |
| **Nutrients per 100 g** | **Substitutes (n=14)** | | **Keyhole (n=7)** | | | **Regular (n=11)** | | | | **P-value*** | |
| Energy (kcal) | 198.5 | (173.8-223.3)^a^ | 148.0 | (146.0-156.0)^b^ | | 241.0 | | (222.0-250.0)^c^ | | <0.001 | |
| Fat (E%) | 57.0 | (52.0-67.0)^b^ | 55.5 | (55.5-57.7)^b^ | | 70.9 | | (69.1-73.0)^a^ | | <0.001 | |
| Saturated fat (E%) | 6.2 | (4.2-23.7)^b^ | 19.5 | (16.7-19.7)^b^ | | 25.8 | | (25.1-28.9)^a^ | | <0.001 | |
| Carbohydrates (E%) | 13.4 | (9.6-19.5)^b^ | 15.1 | (14.1-16.0)^b^ | | 10.0 | | (7.9-10.8)^a^ | | 0.012 | |
| Sugars (E%) | 2.9 | (1.2-3.6) | 1.3 | (1.1-1.8) | | 1.3 | | (0.7-2.1) | | 0.083 | |
| Fibre (E%) | 3.1 | (0.0-5.1)^a^ | 0.0 | (0.0-0.0)^b^ | | 0.0 | | (0.0-0.0)^b^ | | <0.001 | |
| Protein (E%) | 24.9 | (16.7-32.9) | 30.1 | (25.8-30.1)^a^ | | 18.0 | | (17.1-22.8)^b^ | | 0.016 | |
| Salt (g) | 1.8 | (1.3-1.9) | 1.7 | (1.7-1.8) | | 1.7 | | (1.6-1.9) | | 0.954 | |
|  |  |  |  |  | |  | |  | |  | |
| **Mince, median (IQR)** | | | | | | | | | | | |
| **Nutrients per 100g** | **Substitutes (n=13)** | | **Keyhole (n=13)** | | | **Regular (n=13)** | | | | **P-value*** | |
| Energy (kcal) | 195.0 | (157.5-220.0)^b^ | 129.0 | (120.5-146.5)^a^ | | 202.0 | | (200.0-205.0)^b^ | | <0.001 | |
| Fat (E%) | 43.1 | (21.5-54.6)^b^ | 36.0 | (34.4-50.8)^b^ | | 62.4 | | (61.9-64.0)^a^ | | <0.001 | |
| Saturated fat (E%) | 4.4 | (3.0-24.8)^b^ | 16.0 | (14.0-17.2)^b^ | | 28.1 | | (27.9-30.9)^a^ | | 0.001 | |
| Carbohydrates (E%) | 14.6 | (7.0-19.2)^a^ | 0.0 | (0.0-0.0)^b^ | | 0.0 | | (0.0-0.0)^b^ | | <0.001 | |
| Sugars (E%) | 2.3 | (0.9-3.2)^a^ | 0.0 | (0.0-0.0)^b^ | | 0.0 | | (0.0-0.0)^b^ | | <0.001 | |
| Fibre (E%) | 5.0 | (0.0-7.7)^a^ | 0.0 | (0.0-0.0)^b^ | | 0.0 | | (0.0-0.0)^b^ | | <0.001 | |
| Protein (E%) | 36.6 | (30.1-47.1)^b^ | 64.0 | (49.2-65.6)^a^ | | 37.6 | | (35.5-38.0)^b^ | | <0.001 | |
| Salt (g) | 1.0 | (0.6-1.7)^a^ | 0.1 | (0.1-1.0)^b^ | | 0.2 | | (0.1-1.0)^b^ | | 0.002 | |
|  |  |  |  |  | |  | |  | |  | |
| **Meatballs, median (IQR)** | | | | | | | | | | | |
| **Nutrients per 100g** | **Substitutes (n=10)** | | **Keyhole (n=1)** | | | **Regular (n=10)** | | | | **P-value*** | |
| Energy (kcal) | 184.5 | (167.5-214.0) | 156.0 | (156.0-156.0) | | 205.0 | | (181.8-220.5) | | 0.191 | |
| Fat (E%) | 44.4 | (30.5-57.0)^a^ | 50,19 | (50.19-50.19) | | 63.5 | | (60.6-67.9)^b^ | | 0.013 | |
| Saturated fat (E%) | 4.2 | (3.4-5.1)^a^ | 11,54 | (11.54-11.54) | | 24.1 | | (18.9-26.1)^b^ | | 0.001 | |
| Carbohydrates (E%) | 20.1 | (13.6-30.9)^a^ | 12,56 | (12.56-12.56) | | 9.1 | | (7.6-13.5)^b^ | | 0.016 | |
| Sugars (E%) | 2.3 | (1.6-4.4)^a^ | 1,03 | (1.03-1.03) | | 1.8 | | (0.4-2.6) | | 0.195 | |
| Fibre (E%) | 5.0 | (0.0-5.8) | 0.00 | (0.00-0.00) | | 0.0 | | (0.0-0.4) | | 0.063 | |
| Protein (E%) | 26.7 | (20.6-43.5) | 35.90 | (35.90-35.90) | | 23.6 | | (21.9-28.6) | | 0.341 | |
| Salt (g) | 1.5 | (1.1-1.6) | 1.7 | (1.7-1.7) | | 1.8 | | (1.6-1.9) | | 0.111 | |

IQR, Inter Quartile Range
^a,b,c^ Groups with different superscript differ in Kruskal-Wallis pairwise comparison (p <0.05)
* Kruskal-Wallis Test for differences between groups
† IQR missing due to n=2
‡ Differences between Keyhole and Regular in percentiles outside

Table 7 Differences in nutrient content between plant-based substitute products, dairy with keyhole and dairy without keyhole per 100 g
of product, according to sub-categories cheese, milk (unflavoured drinks) and yoghurts.

| **Cheese, median (IQR)** | | | | | | | |
| --- | --- | --- | --- | --- | --- | --- | --- |
| **Nutrients per 100 g** | **Substitutes (n=36)** | | **Keyhole (n=5)** | | **Regular (n=25)** | | **P-value*** |
| Energy (kcal) | 270.0 | (227.3-285.0)^b^ | 268.0 | (237.0-270.0)^b^ | 300.0 | (257.0-351.0)^a^ | 0.006 |
| Fat (E%) | 72.6 | (70.0-80.2)^b^ | 53.7 | (47.5-54.6)^a^ | 69.2 | (66.9-85.4)^b^ | 0.003 |
| Saturated fat (E%) | 63.3 | (36.4-66.4)^a^ | 33.6 | (29.2-35.8)^b^ | 44.3 | (43.4-55.2)^b^ | 0.001 |
| Carbohydrates (E%) | 28.1 | (17.0-32.2)^a^ | 0.0 | (0.0-0.0)^b^ | 1.9 | (0.1-3.9)^b^ | <0.001 |
| Sugars (E%) | 0.0 | (0.0-0.8) | 0.0 | (0.0-0.0)^a^ | 0.4 | (0.0-3.8)^b^ | 0.017 |
| Fibre (E%) | 0.0 | (0.0-0.0) | 0.0 | (0.0-0.0) | 0.0 | (0.0-0.0) | 0.407 |
| Protein (E%) | 0.2 | (0.0-1.4)^a^ | 46.3 | (45.4-52.5)^b^ | 29.1 | (10.0-31.3)^b^ | <0.001 |
| Salt (g) | 2.0 | (1.5-2.3)^a^ | 1.1 | (1.1-1.3)^b^ | 1.2 | (1.0-1.4)^b^ | <0.001 |
|  |  |  |  |  |  |  |  |
| **Milks (unflavoured drinks), median (IQR)** | | | | | | | |
| **Nutrients per 100 g** | **Substitutes (n=41)** | | **Keyhole (n=10)** | | **Regular (n=9)** | | **P-value*** |
| Energy (kcal) | 42.0 | (30.0-54.0)^b^ | 36.5 | (33.0-38.0)^b^ | 43.0 | (41.0-65.0)^a^ | 0.012 |
| Fat (E%) | 40.0 | (22.6-47.7)^a^ | 7.4 | (2.7-13.5)^b^ | 25.7 | (22.2-55.0)^a^ | <0.001 |
| Saturated fat (E%) | 4.7 | (3.7-7.1)^b^ | 5.0 | (2.7-8.0)^b^ | 17.1 | (15.6-35.7)^a^ | <0.001 |
| Carbohydrates (E%) | 46.2 | (24.2-69.6) | 49.7 | (48.3-54.8) | 42.9 | (29.1-43.9) | 0.154 |
| Sugars (E%) | 30.0 | (23.1-43.2)^b^ | 49.7 | (48.3-54.8)^a^ | 42.9 | (29.1-43.9)^b^ | <0.001 |
| Fibre (E%) | 2.5 | (0.0-3.3)^a^ | 0.0 | (0.0-0.0)^b^ | 0.0 | (0.0-0.0)^b^ | <0.001 |
| Protein (E%) | 8.3 | (4.2-16.0^a^ | 40.1 | (37.3-42.4)^b^ | 31.6 | (20.3-34.1)^b^ | <0.001 |
| Salt (g) | 0.1 | (0.1-0.1) | 0.1 | (0.1-0.1) | 0.1 | (0.1-0.1) | 0.911 |
| **Yoghurts, median (IQR)** | | | | | | | |
| **Nutrients per 100 g** | **Substitutes (n=42)** | |  | | **Regular (n=19)** | | **P-value*** |
| Energy (kcal) | 80.0 | (67.5-107.3) |  |  | 86.0 | (71.0-90.0) | 0.379 |
| Fat (E%) | 30.5 | (23.3-58.4) |  |  | 31.0 | (30.0-42.9) | 0.950 |
| Saturated fat (E%) | 5.5 | (3.4-49.9) |  |  | 21.1 | (19.8-28.6) | 0.026 |
| Carbohydrates (E%) | 41.2 | (27.6-59.4) |  |  | 50.0 | (30.6-53.3) | 0.674 |
| Sugars (E%) | 26.7 | (13.9-45.6) |  |  | 47.1 | (30.6-50.6) | 0.003 |
| Fibre (E%) | 2.2 | (1.2-3.3) |  |  | 0.0 | (0.0-0.0) | <0.001 |
| Protein (E%) | 8.1 | (4.9-21.5) |  |  | 17.4 | (16.3-23.8) | 0.007 |
| Salt (g) | 0.1 | (0.0-0.2) |  |  | 0.1 | (0.1-0.1) | 0.826 |

IQR, Inter Quartile Range
^a,b,c^ Groups with different superscript differ in Kruskal-Wallis pairwise comparison (p <0.05)
* Kruskal-Wallis Test for differences between groups in sub-categories cheese and milk; Mann-Whitney U Test for differences between
groups in sub-category yoghurt
